# An Enhanced Distributed Data Aggregation Method in the Internet of Things

**DOI:** 10.3390/s19143173

**Published:** 2019-07-18

**Authors:** Mohammad Hossein Homaei, Ely Salwana, Shahaboddin Shamshirband

**Affiliations:** 1 Internet of Things Laboratory of Iran (Gloriot), Hamedan, Iran; 2 Institute of Visual Informatics, Universiti Kebangsaan Malaysia, Bangi 43600, Selangor, Malaysia; 3 Department for Management of Science and Technology Development, Ton Duc Thang University, Ho Chi Minh City, Vietnam; 4 Faculty of Information Technology, Ton Duc Thang University, Ho Chi Minh City, Vietnam

**Keywords:** data aggregation methods, learning automata, RPL, routing, Internet of Thing (IoT)

## Abstract

“Internet of Things (IoT)” has emerged as a novel concept in the world of technology and communication. In modern network technologies, the capability of transmitting data through data communication networks (such as Internet or intranet) is provided for each organism (e.g., human beings, animals, things, and so forth). Due to the limited hardware and operational communication capability as well as small dimensions, IoT undergoes several challenges. Such inherent challenges not only cause fundamental restrictions in the efficiency of aggregation, transmission, and communication between nodes; but they also degrade routing performance. To cope with the reduced availability time and unstable communications among nodes, data aggregation, and transmission approaches in such networks are designed more intelligently. In this paper, a distributed method is proposed to set child balance among nodes. In this method, the height of the network graph increased through restricting the degree; and network congestion reduced as a result. In addition, a dynamic data aggregation approach based on Learning Automata was proposed for Routing Protocol for Low-Power and Lossy Networks (LA-RPL). More specifically, each node was equipped with learning automata in order to perform data aggregation and transmissions. Simulation and experimental results indicate that the LA-RPL has better efficiency than the basic methods used in terms of energy consumption, network control overhead, end-to-end delay, loss packet and aggregation rates.

## 1. Introduction

The expression “every Internet-connected thing is alive” will be a new rule in the near future. Perhaps, future networks are merely IoT, toward which all investigations will soon be converged. Similar to the procedure of using the Internet by humans; from now on, new devices will be the primary users of the Internet ecosystem. The attention of researchers has been recently devoted to newly emerged technologies for manufacturing scalable IoT. However, the speed of comprehending the IoT framework reduced due to several factors, among which the combination of different devices, secure connections, trust management, and the collaboration between devices and systems are highlighted. Such devices collaborate in order to aggregate, share, and conduct information in a multi-hop manner. The bulk of information continuously generated by IoT necessitates the conversion of aggregated data into intelligence. Such an intelligent environment can play a vital role in data routing of networks [1,2]. The consistent mobility of most IoT nodes leads to the alternative communication between devices, and variable network topology as a result [3]. As a result of such frequent topology variations as well as the limited resources present in today’s IoT devices, routing scheme of such networks is regarded as a significant challenge in the research world [4].

Coverage, sensing, connection, and communication of each node in IoT requires energy and cost expenditures. As IoT equipment is becoming more and more portable and smaller, the energy limitation of such equipment is a significant and challenging issue to be addressed [5]. Thus, prolonging the lifetime of network nodes in order to provide long term monitoring is considered as one of the staple goals of myriads of IoT protocols. On the other hand, as the rate of the generated and transmitted data toward the base station of the network is remarkably significant, the data aggregation procedure in IoT nodes is also costly. The primary goal of data aggregation methods is to collect and classify data packets in an acceptably efficient manner in terms of energy consumption rate, network lifetime, traffic bottleneck, and data purity [6]. The type and approach of the employed data aggregation method varies based on the topology type, communication type, network design, and data generation rate [7]. In data aggregation procedure, if the central nodes do not perform accurately, the base station will not be able to provide an accurate estimation of the received data; all of which result in the efficiency decline of the network. Hence, data aggregation approaches play an important role in monitoring purposes as well as the long term observation of the environment [8]. In this paper, an efficient distributed data aggregation method for IoT is proposed. Notwithstanding dozens of conventional approaches, calculation overhead of the proposed method is negligible. Moreover, as the network traffic varies, the proposed method still makes correct decisions automatically. 

The rest of this paper is organized as follows. System model and related works regarding IoT and data aggregation methods are presented in Section 2. In Section 3, a novel distributed data aggregation method, is proposed. Performance evaluation of the proposed method in simulation and practical environments are respectively illustrated in Section 4 and Section 5. Finally, Section 6 concludes the paper.

## 2. System Model and Related Works

### 2.1. Internet of Things Architecture

**Figure 1:**
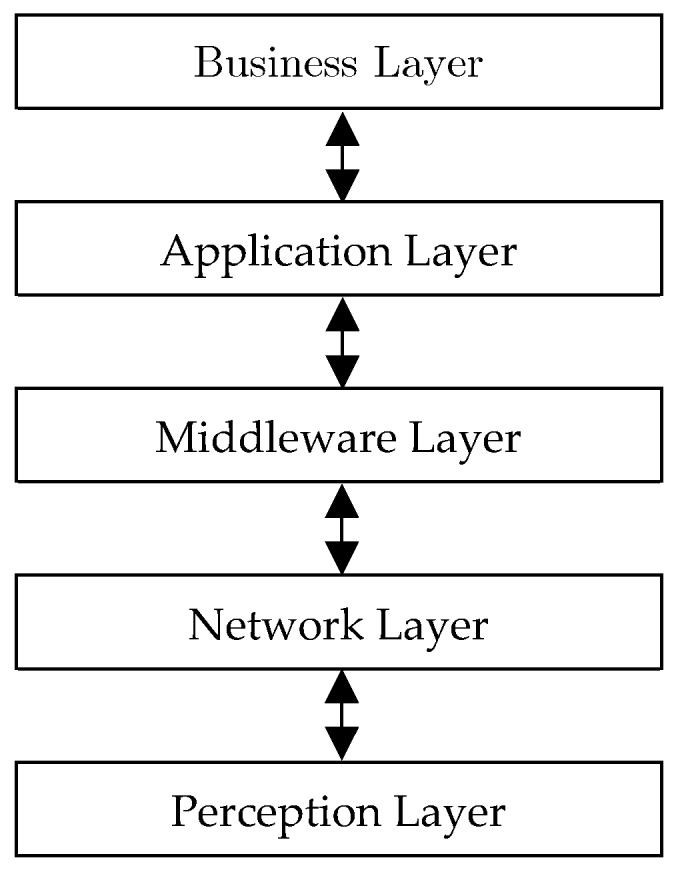
The IoT five-layer architecture [12,14].

Since IoT is to connect a considerable number of heterogeneous things through the Internet, this technology is significantly dependent on the existence of a flexible layer architecture. In other words, IoT is to bridge the real world and virtual world such that dynamic communication is ensured [9]. Therefore, IoT requires a novel architecture in order to obviate the inherent challenges as well as providing acceptable scalability and Quality of Service (QoS) in separate applications [10]. According to the presented architectures in [11,12,13,14], layers of IoT are illustrated in Figure 1.

1. Perception layer: This layer is generally known as the physical or hardware layer, and is particularly allocated to sensors and edge recognizers of a network. In other words, physical and environmental parameters are converted into sensed data in this layer. The output of this layer will be considered as the input of the network layer.
2. Network layer: The main goal of this layer is to provide both direct and indirect communications between all things and IoT equipment, such that all of them are capable of sending and receiving data in the network. Note that the communicative infrastructures managed by network layer include all wireless communication technologies.
3. Middleware layer: The main task of this layer is to combine names and addresses in order to serve other components of the network. Programmers of IoT are endeavoring to connect heterogeneous things under a communicative platform, such that a united concept of network is created and exchanged data in databases can be saved and restored.
4. Application layer: This layer usually merges and evaluates the services provided by other layers. This layer can present high-quality services for responding to the final user’s applications.
5. Business layer: Also known as the management layer of all components of the IoT network, is to analyze and schematize data.

### 2.2. Data Aggregation Strategies in IoT

Data aggregation (DA) is counterproductive to this distributed raw data from various sources within the network. The accumulation in the network has a significant impact on the number of packets transmitted or their length, thereby reducing energy consumption and prolonging network life [15]. The three main scheduling policies for DA are summarized in [16]: (i) Quadruple simple aggregation requires each router to collect all data items periodically and collect them after a period of time Preset. (ii) Periodic dispersal aggregation operates in a similar manner to a simple periodic strategy; however, transferring data as soon as the node receives data from all its children. (iii) Finally, in a moderated accumulation periodically, each node in the aggregate tree adjusts one hour of time-based on its position and sends the result of the conclusion.

**Figure 2:**
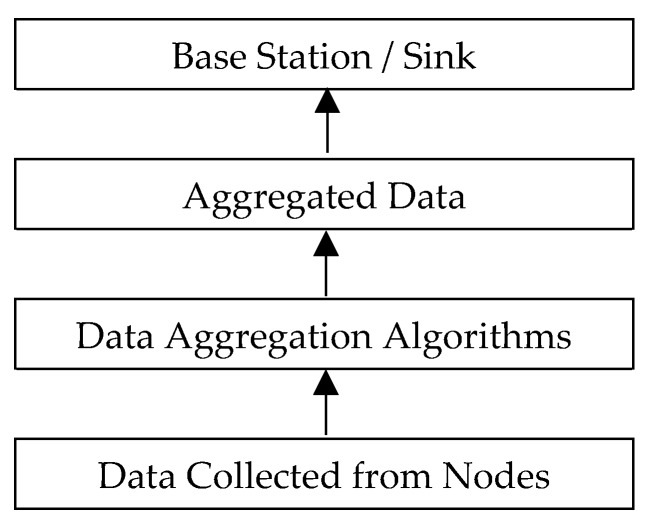
Data aggregation architecture [11].

One of the chief motivations of utilizing data aggregation schemes in low-power and lossy networks (LLNs) and IoT is to decrease energy consumption and increase network lifetime [17]. Since each network node not only has limited capacity to generate, process, and save data; but it is also responsible for data exchange of its neighbor nodes; the necessity of efficient utilization of network resources is axiomatic. Toward this goal, a number of data aggregation methods are proposed, in order to remove the redundancy of unnecessary and digressive data, and reduce communication costs as well [18,19]. The process and architecture of data aggregation in LLN networks and IoT is illustrated in Figure 2

Authors in [20] proposed a three-layer data aggregation infrastructure, called dynamic data aggregation scheme (PDDA) based on priorities for sensor networks because the sensors collect a large amount of redundant data. The proposed PDDA scheme is a hybrid approach that uses clustered and tree-based approaches based on application types. Thus, the proposed PDDA approach achieves energy efficiency and reduces data processing time and overhead at the big data server level. The proposed data aggregation infrastructure has three Layers [21]:

- Data aggregation at the level of the sensors-layer 1
- Data aggregation at the base station (BS)-layer 2
- Data aggregation at the big data Server or NoSQL Server-layer 3 server.

It should be noted that our proposed method in this paper is at the level of sensor nodes to combine effective data with energy and efficient delay. Therefore, the other two layers mentioned in the PDDA method do not fall within the scope of our paper.

**Figure 3:**
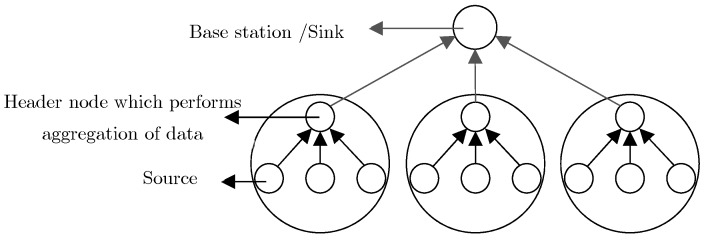
Cluster-based architecture [27].

The important data aggregation mechanisms, as well as their differences, benefits, and weaknesses, are all described in this section. This section is divided into three categories, including cluster-based, centralized and tree-based aggregation mechanisms for IoT:

(1) **Cluster-based mechanisms:** Cluster-based networks can reduce the performance load in terms of reducing the aggregate computation and energy consumption of all nodes [22]. In cluster-based mechanisms, the network environment is divided into different clusters while each cluster comprises a number of sensor nodes [23,24]. In each cluster, one node is selected as the head of all other nodes in a cluster. This will not only decrease the number of transmitted packets, but it also reduces the transmission overhead and bandwidth consumption in transmissions from the cluster to a base station [25]. In [26], a data storage system is presented, which has an efficient functionality in terms of security, scalability, flexibility, and reliability in IoT to be used in the procedure of enormous data analysis. The proposed approach of this research is to present a distributed storage infrastructure providing scalability and reliability in IoT. Multiple data storage in the distributed system provides networks with fault tolerance, reliability, and stability. The general structure of cluster-based mechanisms is depicted in Figure 3.

(2) **Centralized mechanisms:** Another suggested data aggregation method in IoT is termed as the centralized method [28]. In this method, the data existing in all other network nodes along a route is transmitted to only one node. In other words, all nodes deliver their sensed information to a single node, which is superior to other network nodes in terms of hardware features and resources. In this method, the central node generally aggregates several data packets and converts them into a single packet [29]. In [30], an IoT communication platform is proposed, in order to support wireless sensor network nodes activity and proper data delivery rate. In this approach, while the unity of data is conserved, confidentiality and accessibility of the network are taken into consideration as well. However, the complexity of this data aggregation mechanism is not evaluated in practical applications. Authors in [31] presented a distributed service-driven architecture to gather data from multiple nodes in various applications of IoT. This mechanism not only alleviates network traffic, but it can also be employed as a flexible mechanism to share data in disparate programs. However, the main challenge of this mechanism is the low accessibility of the central node in the network. Also, data will be lost if breaks occur in the central node [32].

(3) **Tree-based mechanisms:** Another structure of IoT topology is based on trees. In this structure, tree construction is initiated from the root node (i.e., sink node), and proceeds hierarchically to reach leaf nodes located at the final level. After the formation of network tree, leaf nodes and median nodes start sensing the parameters associated with the application type. Afterward, through the accessible parent, each node begins exchanging and transmitting data to the root node. Nodes placed between the root node and the leaf node act as the collector of the transmitted packets from its children. One of the main goals of the tree-based structure in LLN networks and multi-hop wireless networks is to conserve energy as well as reducing the hidden terminal effect in the network, in that by utilizing multi-hop communication, an acceptable balance is established in energy consumption rate [16,33].

**Figure 4:**
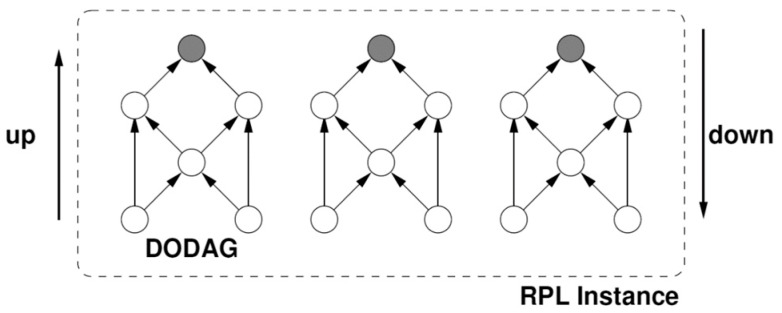
An illustrative network in RPL [37].

The Internet Engineering Task Force group(IETF) has proposed a routing scheme for LLN networks such as IoT and sensor networks, such that Internet Protocol version 6 (IPv6) is extended based on the Routing Protocol for Low-Power and Lossy Networks (RPL) [27], [34]. RPL is an extended distance vector-based protocol for IoT. Routing limitations and challenges in sensor networks, as the most salient subset of IoT, distinguishes it from all other distributed systems [35]. Such limitations affect the whole design of wireless sensor networks, including various protocols and algorithms of other classifications of IoT. This research has focused on the routing scheme of IoT [36]. RPL consists of an acyclic graph with one root per Destination Oriented Directed Acyclic Graph (DODAG). In such a graph, each node acts as the parent node if required; otherwise, it is considered as a child of an accessible parent. The utilization of a point to multi-point approach is another feature of this approach, which is consistent with the applications of sensor networks. As depicted in Figure 4, this protocol creates a DODAG as the root. 

If the personal area network coordinator (PAN-Coordinator) is considered as the root of the directed acyclic graph (DAG), several paths can be established toward the PAN coordinator. However, in accordance with the considered policies in RPL objective function, any kind of loop creation is avoided. RPL can exploit any routing metric to create DODAG. Each node broadcasts a DAG information object (DIO) containing the distance between the node and DAG root in terms of a specific metric (e.g., several hops, link quality, delay or Jitter). Afterward, each node executes a distance vector algorithm, in order to find a set of neighbor nodes which are closer to the root than the node itself.

Such neighbors are the very parent nodes. Additionally, RPL presents a fast route repair mechanism to be utilized if any unstable loop is detected. Although RPL is implemented and completely evaluated in TinyOS and Contiki [5], it is rarely employed on a short MAC duty cycle. As far as the investigations of RPL reveal, RPL is not even evaluated under the operation of active beacon IEEE 802.15.4. A number of recent routing schemes support the multiple route utilization in wireless sensor networks. Authors in [38] propose the method of selecting the best path among all existing paths in order to obviate some of the QoS requirements in industrial applications. To demonstrate RPL more clearly, it is crucial to define the basic principles, based on which the algorithm is proposed. In this regard, by considering an RPL network named *G*, consisting of node set *S* and boundary routers *B* (DODAG concept) the concepts of rank, high-priority parent of DODAG, and root list of DODAG are illustrated as follows [34]:

**Definition** **1.** *Rank: Rank R(u, j) is a criterion of the distance between the node u∈S and DODAG root j∈B. The accurate rank calculation method depends on the DAG objective function (OF). Although the rank calculation falls within the obligations of objective function, nodes’ rank should steadily decline while moving in DODAG toward DODAG destination. This is why rank can be construed as a numerical representative of the location or radius of a node within DODAG.*

**Definition** **2.** *DODAG Preferred Parent (DPP): Suppose node u in G, where the single-hop neighbors set is denoted by N(u), and DPP(u, j) is a limited subset of N(u). For each node v∈N(u) we have v∈DPP(u, j), provided that v is of the minimum rank toward the specified DODAG root j∈B.*

**Definition** **3.** *DODAG Root List (DRL): As mentioned formerly, each node v∈N(u) should send DIO messages in broadcast manner. In GeoRank operation, the location of DODAG root should be included in such messages. Hence, it is supposed that DRL(j) is a saved list of DODAG root location in each node u.*

For each sink node, RPL creates and supports at least one DODAG. According to the predefined specific procedure, this protocol calculates upstream and downstream routes independently; to take advantage of them if the network objective function is fulfilled. Notwithstanding the features and privileges of RPL protocol, this protocol suffers from a kind of unfairness, and unbalanced traffic transmission, since the introduced objective functions in papers support a specific objective function depending on the particular application. As elucidated in [39], one of the recently proposed solutions is the graph degree restriction in RPL network. As mentioned by authors, graph degree restriction is the main principle of discriminating parent node in child node selection. In this approach, the network is modeled by a connected non-directional graph G (V, E).

Constant value k is defined such that k < |V|, where |V| is the number of existing nodes in the network graph. Constant value k, which indicates the constraint of the number of acceptable children, is recognized by each node. In other words, k is the maximum node degree in DODAG. Note that the root node does not obey this constraint. In the constitution procedure of the DODAG, each node v in DODAG selects the optimum parent p, and memorizes a potential set of alternative parents to construct upstream nodes. To implement the graph degree constraint in Bounding Degree RPL (BD-RPL), the principle messages of RPL protocol, like DAO and DAO-ACK, are utilized. A list of variables used in the paper are shown in Table 1.

Due to the tremendous topological changes and the resultant requirement to synchronize and update routing tables, a significant number of control messages are exchanged in most of the protocols proposed for IoT. For the aim of conserving energy in most of the equipment’s with limited resources, such communication costs should be controlled [40]. Authors in [16] focus on the LLN networks protocols, especially RPL, where resource limitations remarkably affect efficiency. A flexible approach, named Adaptive-RPL (A-RPL), a protocol that uses periodic simple aggregation method called Modified RPL (M-RPL) was proposed in order to create and change network conditions through objective function formation framework. In this approach, network data is aggregated along with seeking the root node. The proposed data aggregation method in this protocol is so simple (maximum, minimum, average), such that depending on the application of the data received by the parent node, the maximum value or minimum value is merely sent to its parent. Furthermore, a compatible scheduling model is proposed to optimize the number of control packets in an RPL network. Also, a compatible MAC-based function is introduced in order to determine the message transmission frequency based on the traffic variety and storage degree of the node. 

## 3. Proposed Method (LA-RPL)

In accordance with the demonstration of the previously proposed approaches in Section 2, the proposed method of this paper comprises two objective functions for both network graph creation phase and aggregation-based data transmission and exchange phase. In our proposed method, learning automata-RPL (LA-RPL), according to the first objective function (named OF 1), each parent node is limited to *k* child nodes, where value *k* is determined depending on the network application type. Through the second objective function, namely OF 2, each network node is equipped with a learning automata. According to the status and congestion of the received packets, such that learning automata grants either data aggregation permission or instantly direct transmission permission to the parent node. Subsequent subsections are as follows. The formation of the network graph, based on the objective function OF 1, is illustrated in Section 3.1. The proposed learning automata in the form of objective function OF 2 is presented in Section 3.2.

### 3.1. Network Graph Formation Phase

**Figure 5:**
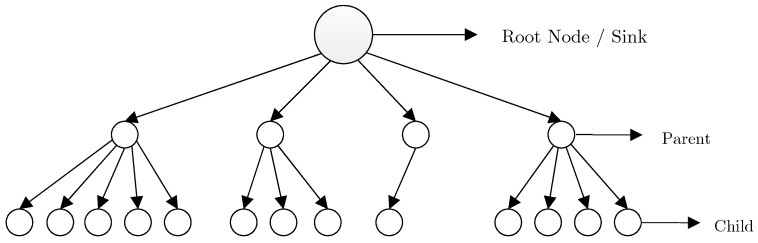
A perspective of RPL graph structure.

RPL graph is generally challenged by the unbalanced work-load and the degree of network nodes. To address this issue, a k degree constraint is determined in order to prohibit each parent node from possessing more than (k) threshold children. It is axiomatic that this approach increases the graph levels. An example of the RPL structure and unbalanced network nodes is illustrated in Figure 5 [27].

**Figure 6:**
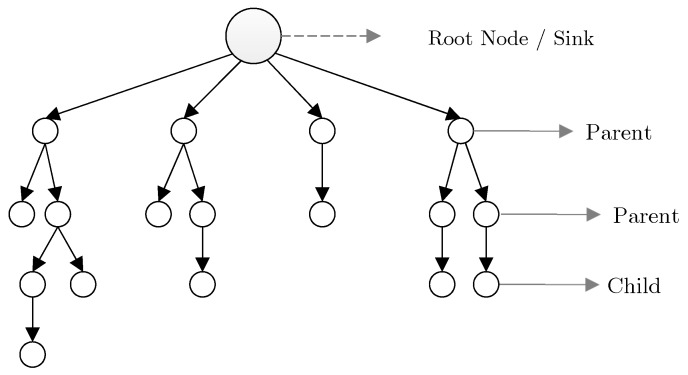
A perspective of a limited degree RPL graph.

For instance, the constraint of degree k = 2 in the connected structure of the network tree is depicted in Figure 6. According to [40] the queueing mechanism of network nodes has limited efficiency on network congestion reduction. However, the act of increasing network graph levels restricts the number of assigned children to a common parent node and reduces the probability of collision and queue overflow in the network as a result.

**Figure 7:**
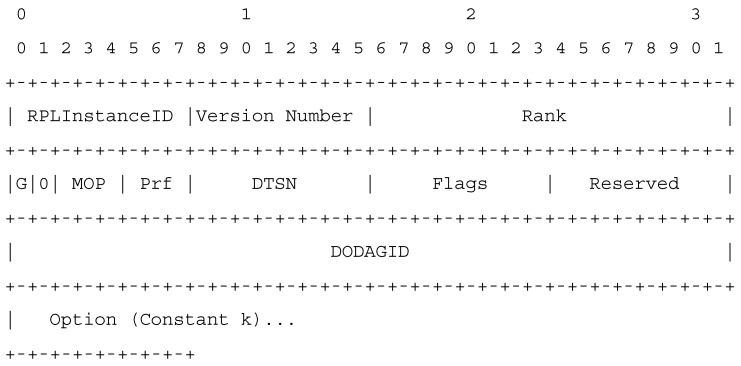
The proposed DIO packet format.

After the placement of network nodes in the environment, the root node determines the type of objective function, based on the priority of the network application type; then, the node broadcasts the degree restriction value (i.e., k constant) through DIO messages throughout the network [27]. Note that the priority of the network application type specifies the targeted end-to-end delay and packet delivery ratio. Having received the DIO message in the first level, each child node investigates the value of graph degree restriction (written in the Options field of DIO message) to verify whether or not this value exceeds the determined threshold. This value must not exceed the determined threshold. We have used this field in the DIO control message to specify graph and threshold. In other words, this field has not added any overhead to the network but has been able to inform one of the target network functions in the formation of the graph to the members of the graph. The parent nodes are aware of the amount of k in the network that cannot send to children more than the threshold *k* of the DAO-Ack message on admission as a child. The proposed structure for DIO message is presented in Figure 7.

In this approach, the number of transmitted (i.e., relayed) packets in nodes placed near the sink node is not changed. However, the collision and packet loss rate decreases, which yields in the reduction of network energy consumption. According to our proposed method, in each node other than the root node, these steps are followed:

1. As soon as node *v* selects its optimal parent (*p*) from DODAG, node *v* assists *p* through sending a DAO message, in order to construct the downstream routes.
2. Since *p* may receive DAO from different children, this node investigates the number of requests at the moment of receiving a DAO. *p* adopts node *v* as its child and adds the existing route to *v* into its routing table, provided that the number of accepted parental requests (including the request of node *v*) does not exceed *k*. By doing so, *p* informs *v* about acceptance of the request through sending a DAO-ACK to *v*. On the other hand, if the number of existing children of *p* exceeds *k* value, *p* denies the request of *v* and informs it through sending a DAO-ACK.
3. Having received the DAO-ACK message, node *v* creates the upstream route to *p* in order to stop the procedure of DAO allocation and confirmation, provided that DAO-ACK is an accept confirmation. However, if the DAO-ACK includes a denial notification, *v* selects another proper parent *p’* from the available parent set, and sends a DAO message to *p’*.

### 3.2. Data Aggregation Scheme Based on Learning Automata

As a novel research topic, the learning mechanism of alive organisms classifies into two general categories. The first category deals with recognizing the learning principles of organisms and the relevant stages. The second category deals with presenting a methodology of placing such principles in a machine. Learning is defined as the occurred changes in a system efficiency based on the former experience. One of the essential features of a learner system is the ability to improve the performance of itself with the passage of time. To put in mathematical explanation, the main objective of a learner system is to optimize a task which is not entirely recognized. Therefore, one of the approaches of this problem is to decrease the objectives of learner system into an optimization problem defined on a set of parameters; the aim of which is to find the set of optimal parameters. A Learning Automata considered as an abstract object with a finite number of operations. Learning Automata operates through choosing one operation from the operation set and applying that operation to the environment. The applied operation is evaluated by a random environment, and the learning automata employs the environmental response to choose its next operation. During this procedure, the automata learn to choose the optimal operation. How to utilize the environmental response of the former operation in selecting the next operation is specified by the learning algorithm of the automata [41]. A learning automata consists of two main components [42]:

- Random automata with a limited number of operations and a random environment communicating with the automata.
- The learning algorithm through which the automata learns the optimal operation.

**Figure 8:**
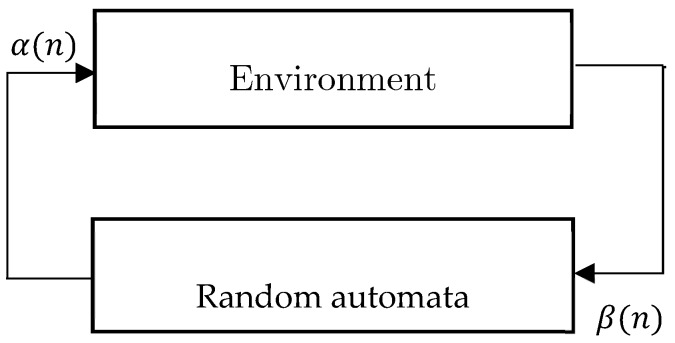
Stochastic Learning Automata [41].

A random automata defined as the fourfold set LA≡{α, β, p, T}, where $\alpha \equiv \left\{\alpha_{1},\;\alpha_{2},\;\ldots ,\;\alpha_{n}\right\}$ is the set of automata’s operations (n denotes the number of automata’s operation), and $\beta \equiv \left\{\beta_{1}\;,\;\beta_{2}\;,\;\ldots ,\;\beta_{m}\right\}$ is the input set of automata. The environment is denoted by the fourfold set of E≡{α, β, c, d}, where $c\equiv \left\{c_{1},c_{2},\;\ldots ,\;c_{n}\right\}$ is the set of penalty probabilities and $d\equiv \left\{d_{1},\;d_{2},\ldots ,d_{n}\right\}$ denote the automata’s bonuses. The environment input is one of the *n* selected operations of the automata. The output (i.e., response) of the environment to each operation *i* is denoted by *βi*. If *βi* is a binary response, the environment is denominated as *P-model*. In such an environment, βi(n)=1 is construed as the unfavorable response, or failure; and βi(n)=0 is considered as favorable response, or success. Set *c* denoting the penalty (failure) probabilities of the environment responses is defined

(1) $$c_{i}=Prob\left\{\beta \left(n\right)=1|\alpha \left(n\right)=\alpha_{i}\right\},\;i=\left\{1,2,3,\ldots n\right\}$$

where the probability of receiving an unfavorable response from the environment is denoted by $\alpha_{i}$. Note that $\alpha_{i}$ values are unspecified, and it is supposed that all values of $c_{i}$ have a unique minimum value. In the same way, the environment can be demonstrated as a set of bonus (success) probabilities (i.e., {$d_{i}$}) where $d_{i}$ denotes the probability of receiving favorable a response from operation $\alpha_{i}$. The relation between the random automata and environment is shown in Figure 8. This set as well as the learning algorithm are denominated as *Stochastic Learning Automata*. In a similar manner, the stochastic learning automata can be demonstrated by the fourfold set LA≡{α, β, p, T}, where $p=\left\{p_{1},\;p_{2}\;,\;\ldots ,\;p_{n}\right\}$ is the border of the probabilities of automata’s operations, and T≡p(n+1)=T[α(n), β(n), p(n)] is the learning algorithm.

If operation $\alpha_{i}$ is selected in the $n^{th}$ step; then, in the ${\left(n+1\right)}^{th}$ step we have:

The favorable response from the environment is

(2) $$P_{i,j}\left(k+1\right)=\{\begin{array}{c} P_{i,j}\left(k\right)+\alpha \left(1-P_{i,j}\left(k\right)\right),\;i=j\\ P_{i,j}\left(k\right)\left(1-\alpha \right),\;\forall j\neq i \end{array}$$

The unfavorable response from the environment is

(3) $$P_{i,j}\left(k+1\right)=\{\begin{array}{c} P_{i,j}\left(k\right)+\left(1-\beta \right),\;i=j\\ \frac{\beta }{r-1}+P_{i,j}\left(k\right)\left(1-\beta \right),\;\forall j=i \end{array}$$

It is worth stating that set α includes the outputs (i.e., the operations) of automata. In other words, the automata selects and applies one operation among all *r* operations existing in this set in each step. Note that the input set *β* determines the inputs of the automata [42].

Having created the network graph, network nodes execute the proposed objective function OF2. Each parent node starts either aggregating data or instantly sending data in a recent time slot. The act of data aggregation is performed by each parent node such that the next-step action is selected according to the environmental received feedback. Note that the proposed system acts in a distributed manner; such that if a parent node in lower layers aggregates some packets, such packets are not aggregated in higher layers, to avoid the multi-step aggregation and the resultant unacceptable imposed delay on data packets. In order for data aggregation mechanism to perform efficiently, each sensor node is equipped with learning automata. Learning automata is a decision-making system selecting an existing operation in the upcoming round according to the environmental received feedback. Learning automata includes two phases: selecting phase, and learning phase. In the selecting phase, based on the environment feedback, decisions are made concerning the upcoming rounds toward improving the current status about previous steps [43].

(1) **Selecting Phase:** All of the sensor nodes have an aggregation label (*lbl_indicator*), which is initialized to 0 at the beginning. When a sensor node plays the role as an aggregator, the value of this label changes to 1. In the routing procedure proceeding data reception, each node acts as an aggregator with probability $P_{Agg}$ and, accordingly, acts as an ordinary node with probability $1-P_{Agg}$ to prepare and send the received data toward the root node. Upon the activation of *lbl_indicator* (i.e., changing the value to 1), the node waits for t seconds to receive more data packets. Note that t is a constant value for all of the nodes acting as an aggregator. After the passage of t seconds, all the received data is aggregated into a single data packet, by means of the function *F* (Aggregation). Afterwards, this single packet is routed. At the beginning, all nodes have the same $P_{Agg}$. However, through the repetitions of the algorithm and the reception of reinforcement signals from the environment, this probability changes.
(2) **Learning Phase:** In the learning phase, a learning automata employed in the internet of things network as a distributed factor. Each node, as a learning agent, is equipped with learning automata comprising two different operations. The concepts and parameters of such a learning automata are as follows:
  - **Agent:** Each sensor node acting as an independent learner is known as an agent. In other words, the action of learning agent has no effect on other learning agents.
  - **Action:** Each agent can act as an aggregator or an ordinary node.
  - **Reinforcement Signal (RS):** The number of data packets received by node *j* during time duration *t* (also known as *input degree*).

(4) $$Rate_{j}=P_{AN}+P_{AM}$$

where $P_{AN}$ is the number of data packets aggregated by previous nodes throughout the routing, and $P_{AM}$ is the number of data packets not being aggregated by previous nodes throughout the routing.

(5) $$RS=1-\frac{1}{Rate\;j}$$

If S>Threshold δ, then node *j* is rewarded. Otherwise, the node is given a penalty. If a reward is received by the node, $P_{Agg}$ varies as

(6) $$P_{Agg}=P_{Agg}+\alpha \times R\times \left(1-P_{Agg}\right),$$

And if a bonus is received by the node, $P_{Agg}$ will be as

(7) $$P_{Agg}=\left(1-\beta \left(1-R\right)\right)P_{Agg},$$

where *β* is the penalty coefficient, *T* is the reward to penalty ratio, and the impact of coefficients for the total number of (plain or aggregated) received packets in node *j* during time duration *t* is

(8) $$N_{AP_{j}}=\sum i=1degree\;N_{PK_{i}}$$

where $N_{PK_{i}}$ is the number of aggregated data packets in *i* for node *j*, and the impact of coefficients (reward/penalty) is as

(9) $$T=1-\frac{1}{N_{AP_{j}}}$$

**Figure 9:**
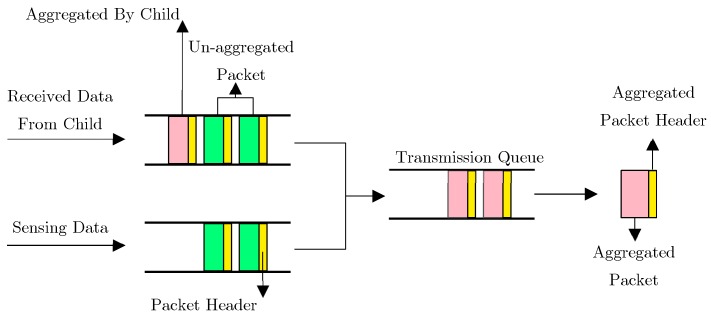
The diagram of data aggregation mechanism in each parent node.

In Figure 9, the status of a node in the network is displayed with the RPL structure. In this figure, depending on the role of the node on the network, either the child node or the parent, one or both types of input can be adopted. For example, for a leaf node, there is only a data sensing unit and packet formation, and in the parent node, in addition to the sensor segment, there is also a unit of message reception from the children. Therefore, the parent node in the proposed method, depending on whether the package received from its child has already been aggregated, can be done with the functions of the automata to create and manage the Transmission Queue. Finally, the outgoing packets from each parent node are packets that once have been aggregated.

**Figure 10:**
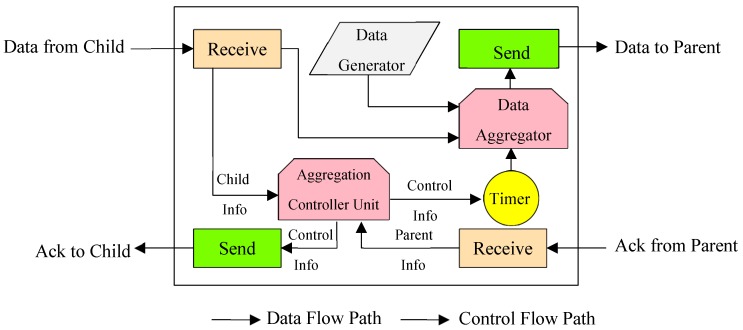
The diagram of data communication and internal control messages in a typical node.

In Figure 10, the internal communication diagram of each node is displayed in the RPL network. In this form, the Data Generator unit has the task of sensing the physical environment and data generation that transmits its output to the aggregation node in the node. The control unit for collecting information from the child nodes manages the information received from the parent and acts on the child to send the message to the data sending unit aggregated to the parent or to send the Ack message. It should be noted that all the units mentioned use their internal timer.

**Figure 11:**
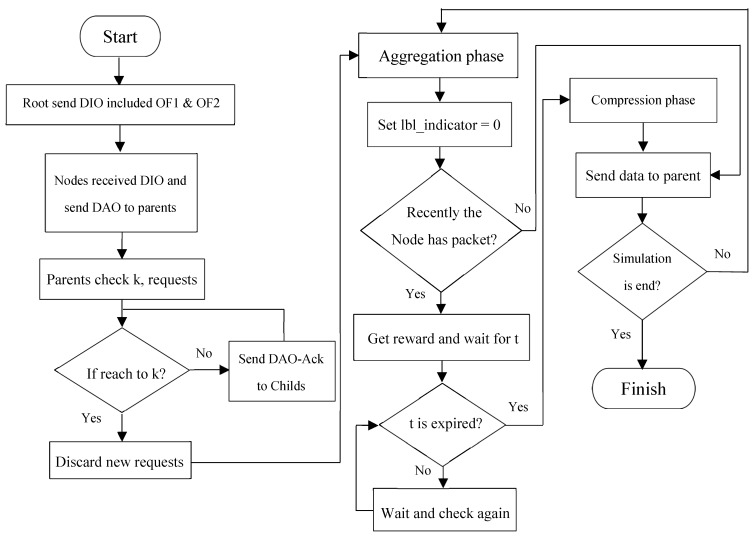
General diagram of the proposed method.

In Figure 11, a general diagram of the proposed method is presented. As shown earlier, the root node uses two objective functions, one to two, to send a DIO message and form a network graph. In the second step, network nodes send the root node after receiving the DIO message to send the DAO message requesting a root subscription. At the first level, there is no limit to the number of children for root nodes. The parent nodes that are rooted are forwarding the DIO message received from the unit by updating the open rank. At this point, each parent node can accept k child numbers and will not respond to requests that exceed the threshold. This process is done to the extent that the network graph is complete. In the aggregation phase, each label has a label (zero). Given that the parent node closed recently? Retrieved with a learning automata system and will wait for t seconds. This will have a greater chance of getting future packs. Otherwise, he will receive a fine and send the packet directly to his parent. The parent node, in the event of expiration, t submits to the aggregation of the data received and its production data, if any, and sends the packet to the parent. In order to avoid multiple aggregations of a packet at different levels of the network graph after the package is first combined, the label will change to 1.

## 4. Performance Evaluation

In order to evaluate the performance of the proposed method and compare its performance with those of base approaches, Contiki Operating System and Cooja emulator were utilized [44]. Contiki is an open source operating system for simulating IoT, which enables us to provide communication between low power and low-cost microcontrollers through the Internet. Additionally, using the embedded tools in the core of Contiki Operating System, this OS provides the implementation of complex wireless networks. Contiki adapted for the hardware which is simultaneously constrained by the memory, power, processing capability, and communication bandwidth. A Contiki-based system usually requires resources such as a kilobyte-ranged memory capacity, a milliwatt-ranged power, a several megahertz-ranged processing frequencies, and hundreds of kilobits per second bandwidth. Such class of hardware includes a wide range, such as common embedded systems to old computers [45,46]. Note that while alluding to an emulator, we imply a software or a hardware system which acts positively close and similar to a real system; such that while utilizing such a system, it is usually supposed that a real system is being utilized. However, it is worth mentioning that the implementation procedure of simulators are entirely different. In other words, simulators do not exactly follow the rules and dealings of a real system. Rather, they have specific rules, some of which may hardly occur in a real, non-simulated system. In the present paper, Cooja emulator was employed, in order to model the proposed methods as well as the base method on the Contiki open source operating system.

**Figure 12:**
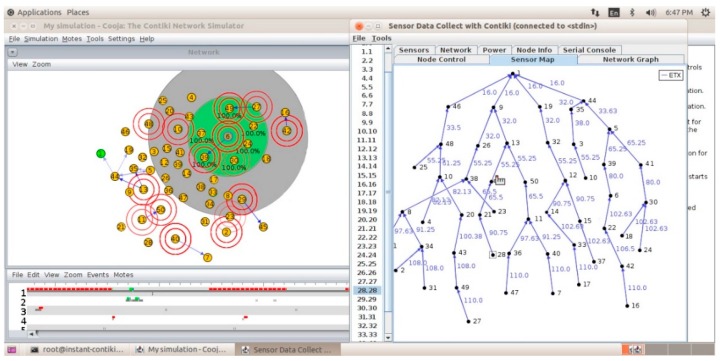
The way to form of the graph with the degree of limitation k = 2 has been presented in Cooja simulation environment.

In both scenarios, there is only one sink node, and 49 sensor nodes are randomly distributed with a 10 m × 20 m in the indoor and outdoor area. The simulation was carried out using 2.66 GHz Intel processor CoreI5 with 8GB RAM on Ubuntu OS. A screenshot of the COOJA simulator environment is shown in Figure 12, which shows the relationship between the nodes of Gloriot. As it is known, all network nodes using DIO and DAO and DAO-Ack messages try to form an enhanced RPL graph with a fixed constraint *k* = 2.

### 4.1. Test Settings

In order to investigate and evaluate the performance of the proposed method, a set of sensor nodes were employed, all of which were produced by our research group (Internet of Things Laboratory of Iran). The designed hardware, which is made in accordance with the specifications elaborated in Table 2 is commercially known as GLORIOT. This test was carried out in Cooja emulator environment and real feedbacks, in order to take test precision into consideration as well.

### 4.2. Energy Consumption Evaluation

IoT nodes commonly use batteries; therefore, the energy source of such nodes are the indispensable factor for their persistent survival and activity in network environment. Regarding this issue, the network energy consumption is measured through two approaches named real energy consumption and nominal energy consumption. In fact, these two measurement approaches are the energy consumption values in real world implementation and Cooja emulator environment respectively. The first solution to calculate the energy consumption rate in milli-Joule scales is modeled as [47,48]:

(10) $$Energy\left(\mathrm{mJ}\right)=\frac{(T_{x}\times 19.5\;mA+R_{x}\times 21.8\;mA+CPU\times 1.8\;mA+LPM\times 0.0545)\times 3V}{4096\times 8}$$

where $T_{x}$ and $R_{x}$ respectively represent the consumed energy in each transmission and reception occurred in a node. In addition, the power consumption level (in milli-watt hour units) in network nodes is calculated through Equation (10) as:

(11) $$Power\left(\mathrm{mW}\right)=\frac{Energy\left(\mathrm{mJ}\right)}{Time\left(s\right)}$$

**Figure 13:**
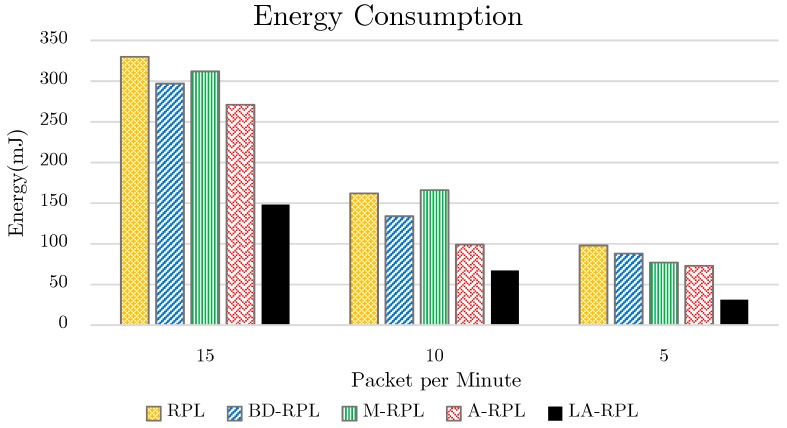
The results of energy consumption evaluation, while network traffic is set to 5, 10, and 15 packets per minute.

The energy consumption diagram of the proposed method as well as those of base RPL approach, bounding degree (BD-RPL), modified RPL (M-RPL) and adaptive RPL (A-RPL) version is depicted in Figure 13. It is obvious from Figure 13 that the decrease of exchange rate as well as the increase of available time of network nodes not only have abated network congestion, but have reduced the number of required efforts for data exchange as well. Accordingly, the proposed method consumes less energy as compared to the base approaches; hence, longer network lifetime is provided by this approach.

### 4.3. Control Overhead Evaluation

According to the assertions in Section 2 of this paper, a relatively high percentage of network activity time is spent on environmental control message exchanges, in as much as each connection or communication in RPL mechanism requires the transmission of control packets. Furthermore, due to the utilization of wireless communication medium, the collision rate (i.e., signal collision) of nodes in a tree-structured network in non-extensive environments is relatively high. This is why the precipitation of reaching the steady state procedure in a network graph, prohibition of Lossy communication, and reduction of network communications through aggregating multiple packets in one packet, will desirably reduce the number of efforts required for accessing the medium. Note that another cause of signal congestion in a RPL tree is the usage of multicast and broadcast messages throughout the network. 

**Figure 14:**
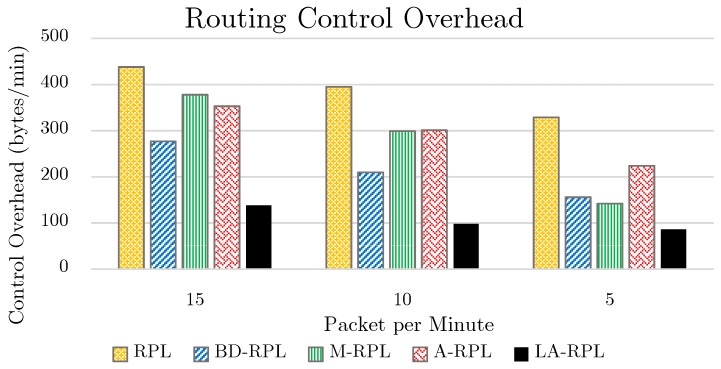
The results of routing control overhead evaluation, while network traffic is set to 5, 10, and 15 packets per minute.

In other words, according to Figure 14, our proposed method has increased the medium access probability for adjacent nodes; in that this method reduces not only the required medium access time but also the number of back-offs occurred in the medium access control layer. The criterion number of control packets in a network is appreciably dependent on the traffic rate (i.e., the number of packets generated in the time unit) as well as on the changes that have taken place in the graph topology structure. In the real world, numerous factors can intensify this procedure; such as noise, signal error in an indoor environment, signal distortion and attenuation, and similar drawbacks preventing the high-quality signal from reaching the intended place. The reduction of RSSI rate is commonly derived from the aforementioned factors, all of which are directly related to the network decisions. Due to the expensive costs of detection, restoration, and recognition of signals in hardware implementations; the message retransmission approach is proposed.

### 4.4. Average Path Length Evaluation

In this test, network performance is investigated through different link successful transmission rates. In particular, link successful transmission rate among nodes varies between values 0.3, 0.5, and 0.7. In other words, the higher successful transmission rate a link has, the higher child-to-parent accessibility is provided, and the fewer number of efforts are required for data transmission. Consequently, as the successful transmission rate of a link reduces, the number of efforts made by a typical child in order to connect to its corresponding parent increases. This leads the connection and communication between child and parent to be more problematic; all of which can culminate in either link failure or consecutive child parent session failures. In this test, which is known as the average path length test, the average number of traversed hops between the leaf node and the root node is indicated. 

**Figure 15:**
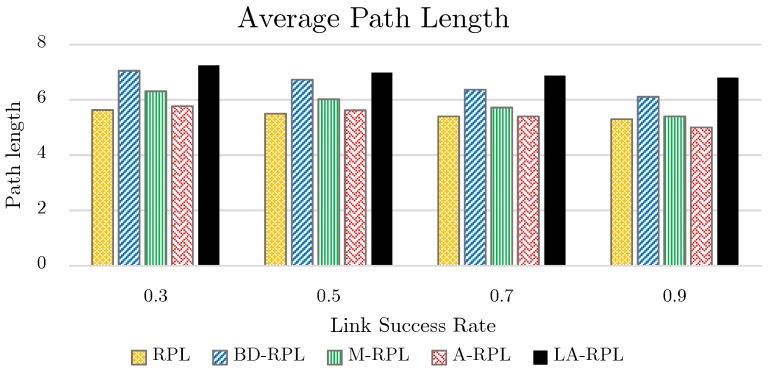
The results of average path length evaluation, while the successful transmission rate of links varies between 0.3~0.9.

Since the primitive criterion for network graph formation in the basic RPL approach is defined as the hop-count distance from the sink node, the values of average path length in this approach is lower than other approaches. In BD-RPL, similar to our proposed method, the number of hop-counts to the root node has increased, in that nodes’ degrees have been restricted. Through the utilization of a combinational objective function as well as the consideration of hop-count parameter in graph formation, the tree height in M-RPL and A-RPL approach has been decreased. In the proposed method of this paper, as a result of degree restriction, the network tree height has been increased. As a result, the more successful transmission rate of link increases, the less average path length; as illustrated in Figure 15. 

### 4.5. Upward Average Delay Evaluation

**Figure 16:**
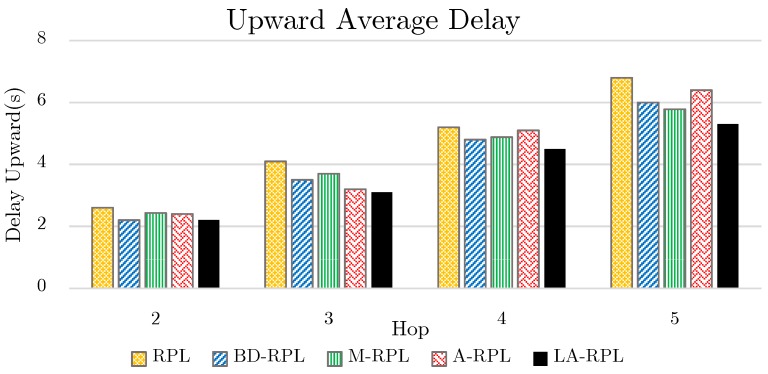
The comparison of average upward delay toward the sink node, while the number of traversed hops varies between 2~5.

The upward consumed time for a packet to reach the sink node is known as a function of the distance to the sink node. According to Figure 16, as anticipated, due to the degree restriction, the upward delay in reaching the sink node in the proposed method is less than those of other approaches. The reduction of network nodes overflow rate as well as the aggregated packet transmissions has brought about a trade-off; such that the slight impact of packets aggregation delay is evident in the network.

In RPL, M-RPL and ARPL approaches, due to the occurred congestion on parent nodes, the delay of seeking the root node is gradually increased. However, as compared to BD-RPL, the degree restriction in the proposed LA-RPL method has caused some time duration to be consumed for aggregation, as well as the common existing packet reception delay, processing delay, transmission delay, and propagation delay. On the other hand, since the packet transmission delay is longer than the aggregation delay, less total time is consumed for packet transmissions by the proposed method in comparison with BD-RPL.

(12) $$AD=\frac{\sum_{i=2}^{n}(D_{Process}+D_{Queue}+D_{Aggergation}+D_{Trans}+D_{Propagate})}{\sum_{i=2}^{n}\left(Hop\right)}$$

In the proposed method, $D_{\mathrm{Aggregation}}$ is a nonzero value. This value is equal to zero for all other approaches.

### 4.6. Network Warming Time Test

**Figure 17:**
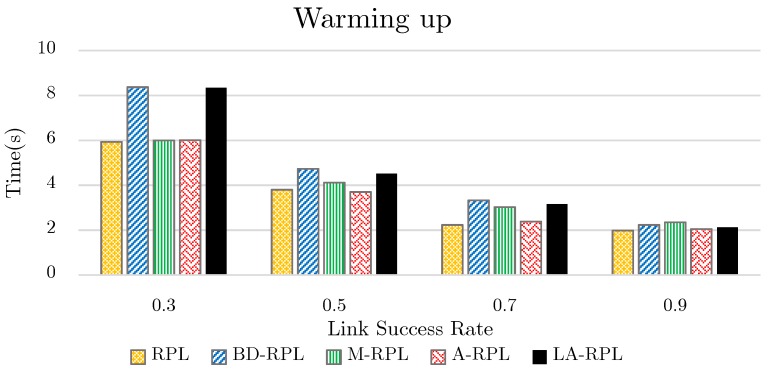
The comparison of warming time in network graph while the successful transmission rate of links varies between 0.3~0.9.

In each RPL network graph, the root node transmits a start message, which is an rudimentary DIO message. Afterward, the network requires an opportunity to create a network graph. As shown in Figure 17, despite former favorable results of the proposed method, this method requires longer network warming time for first time. The warming time of the proposed method consists of the restriction of the number of children assigned to the parent node and amalgamate it into DIO message format, as well as the priority comparison among child nodes for selecting the best parent among all available parents. This procedure requires more incipient exchanges as compared to the base approaches like RPL, M-RPL, and A-RPL. The degree restriction of parent nodes increases the number of levels and effort steps passed by child nodes for the sake of parent node selection; therefore, the height of the network tree has been increased. This approach has been regarded as desirability cost and routing management of network nodes, and, following the inevitable existence of both privileges and deficiencies in each proposed IoT protocol, this can be alluded to the cost of the proposed method. Also, the effect of the successful transmission rate of a link on the warming time also indicates the efforts made by nodes, in order to create the network graph. 

According to Figure 17, in the proposed method of this paper, as the successful transmission rate of links decreases, the waiting time for the creation of a network graph is increased up to four times. However, in RPL, M-RPL, and A-RPL approaches, this increasing trend has been up to at most three times. This observation highlights the significance of the link quality in the proposed method and BD-RPL approach. 

## 5. Performance Evaluation in Practical Tests

**Figure 18:**
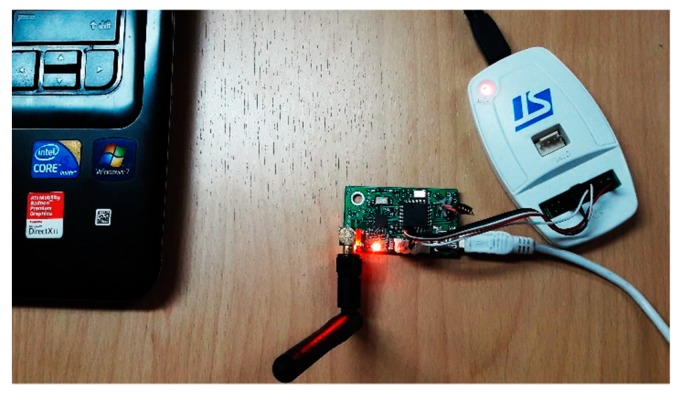
A perspective of the designed sensor node accompanied by ST-Link interface.

For the sake of performance evaluation of the proposed method, some practical tests performed in addition to the previously presented simulation tests. Note that practical tests executed on the same designed hardware with the same conditions. A perspective of the designed sensor nodes in IoT Laboratory(Gloriot) illustrated in Figure 18.

**Figure 19:**
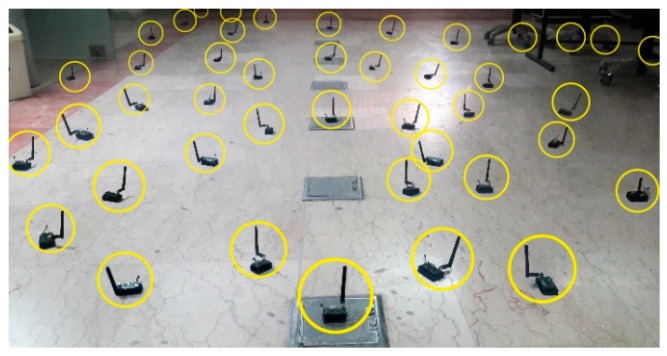
A perspective of nodes distribution in an indoor environment.

**Figure 20:**
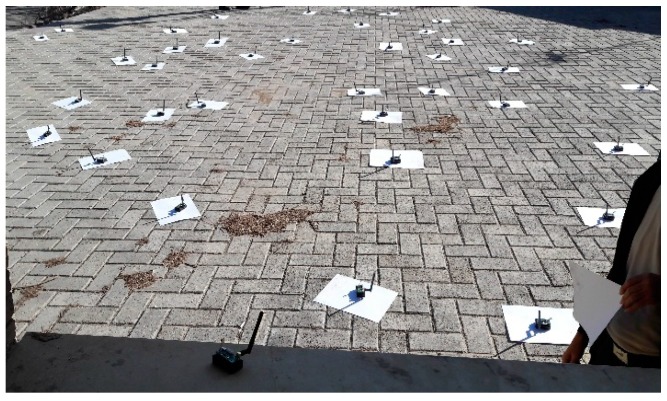
A perspective of nodes distribution in the outdoor environment.

Two network scenarios (Indoor and Outdoor) selected to evaluate the performance of the proposed scheme Learning Automata RPL (LA-RPL). Similarly, the distribution of nodes in indoor and outdoor environments are shown in Figure 19 and Figure 20, respectively.

### 5.1. Routing Packets and Loss Packets and Aggregation Evaluation

In this test, in order to evaluate the performance of the proposed method and investigate its success in the delivery of produced data packets throughout the network, the difference between the network nodes’ transmitted packets and root node’s received packets postulated as the evaluation criterion. The number of packets being retransmitted because the noise is not calculated, withstands the fact that the connection type of this test considered as user datagram protocol (UDP). 

Table 3 shows the number of packets sent/receive/drop, packet delivery ratio (PDR), and Aggregation rate for all evaluated methods are in two indoor and outdoor environments. RPL protocol shows the packet delivery ratio from indoor (91%) and outdoor (95%) environment in the network perspective. Due to the use of graph-grade constraints and the formation of a balanced graph, the delivery rate has risen to 96% in BD-RPL protocol. Using a single objective function in M-RPL method, the percentage of packet delivery ratio (PDR) reaches to 93% in an indoor environment and 95% in outdoor space. The A-RPL method has performed more efficiently than expected in its predecessor’s expectations and reach to 96% of the packets in the network in both indoor and outdoor space, but the proposed method has not yet been able to overtake it. According to the results of the proposed LA-RPL approach, around 98% of packets sent to the network reached in an indoor environment.. On the other hand, the PDR reaches 99%. Thus, the reliability of the packet delivery in the network in the proposed method is far higher than the comparable methods.

Based on results in Table 3 noted that the packet aggregation rate in RPL and BD-RPL is zero because both methods have not any aggregation process in their protocol. In M-RPL aggregation rate is 6.78 and 9.83 in the indoor and outdoor environment respectively, but these rates in A-RPL are increased about 11.03 and 13.26 in each experimental environment. Finally in the proposed method (LA-RPL) aggregation rate is 34.11% in the indoor environment, and 37.3% in the outdoor environment. In other words, for each hundred transmitted packets in the network, approximately 34 packets in the indoor environment and 37 packets in the outdoor environment are received by the root node in an aggregated format.

### 5.2. Average Power Consumption Per Node

**Figure 21:**
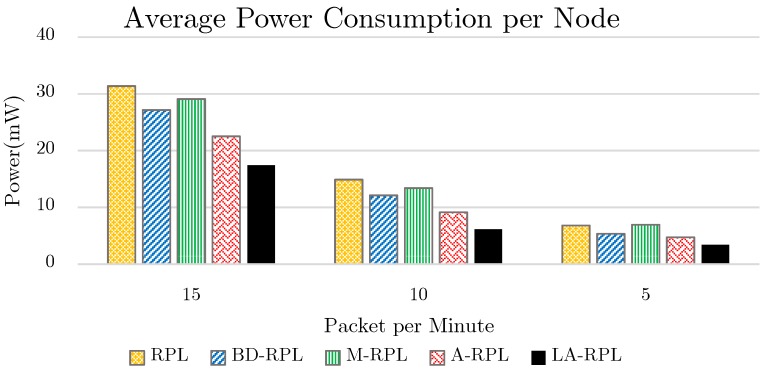
Comparison of average energy consumption of each node, while the traffic rate is 5, 10, and 15 packets per minute.

This test is allocated to the investigation of the average energy consumption for each network node. By the acquired simulation results presented in Figure 13, it is observed through this practice test that the energy consumption of network nodes is reduced. According to Figure 21, the average energy consumption of each network node for traffic rates 5, 10, and 15 packets per minute is respectively 3.37, 6.14, and 17.4 milliwatts.

### 5.3. The Average Number of DIO Control Packets while Topology Changes

**Figure 22:**
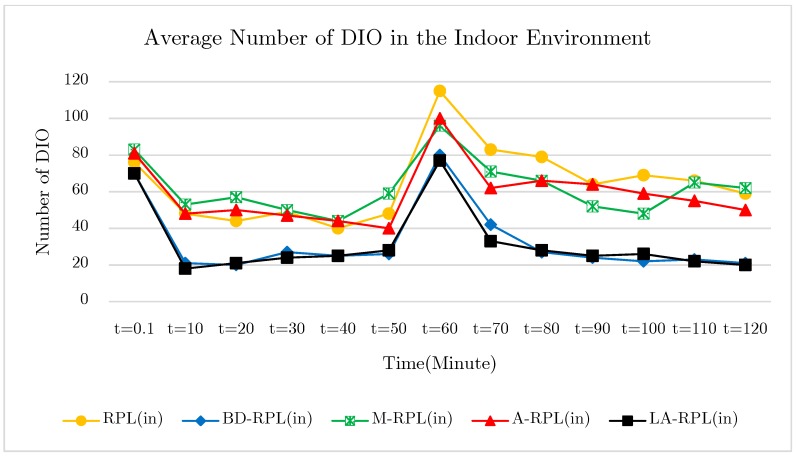
The diagram of trickle timer control of DIO message in indoor environments.

The main objective of this test is to investigate the extent to which the proposed method is effective to preserve trickle timer; such that the transmission of unnecessary DIO messages are avoided. In other words, to what extent the creation of steady network graph can reduce the control overhead, and increase the available time of nodes as well. In the proposed method and BD-RPL, through providing a balance in the network graph, the degree restriction of parent nodes prevents the occurrence of congestion; therefore, the rate of DODAG Information Solicitation messages (DIS) and network instability is reduced in comparison with the base RPL, M-RPL, and A-RPL approach. In Figure 22, the average number of DIO messages was published in the network in the indoor environment over five times and averaged over time.

**Figure 23:**
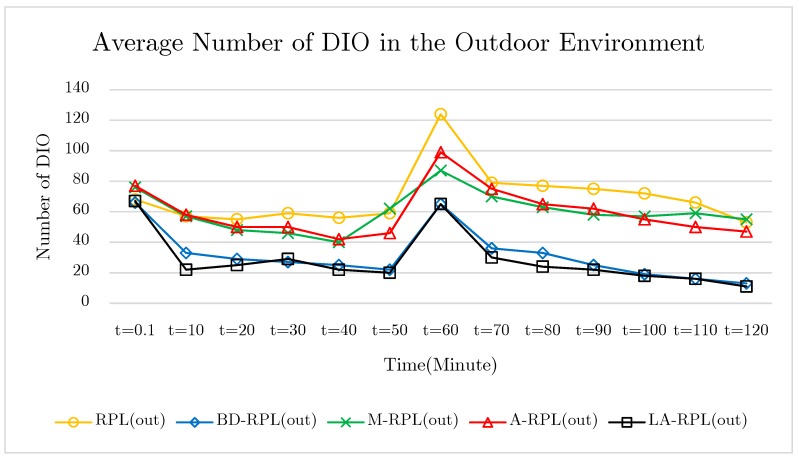
The diagram of trickle timer control of DIO message in outdoor environments.

This is mainly since, despite the existence of upper-threshold recognized instability in the network, a reset trickle timer that culminates in the transmissions of DIO messages, as well as some changes in the network graph are essential. An important note in this test is that the location of five nodes changed after the passage of 60 min from the initial start time. This is done to investigate and compare both approaches in terms of both the speed and the cost of restoring network graph in indoor and outdoor environments. According to the results of the test, the number of published DIO messages in the outdoor environment network is lower than the indoor environment in the proposed method of LA-RPL and BD-RPL, which indicates the stability of the network graph in these two protocols. Also, in the M-RPL and A-RPL methods, due to the lack of focus on the grid structure, there was no systematic and accountable management of the DIO message. The results of the five repetitions of the outdoor test are shown in Figure 23.

## 6. Conclusions

Due to the significance of communications among nodes as well as the topology and data packets’ transmission method in wireless sensor networks, specifically in the Internet of Things, this research investigated the state-of-the-art proposed methods and presented a novel solution for the mentioned issues. According to the presented documentaries in this research, routing approaches in IoT extended facets, such that it was highly dependent on the hardware, software, and the embedded operating system leaded to a number of various challenges. Routing efficiency of a destined source and destination pair were remarkably affected by issues such as computational overhead, algorithmic complexity, security, reliability, hardware fault tolerance, data error, and so forth. Such challenges were so wide-ranging and relevant to the cross layer issues that exceed the scope of this research. This paper focused on the reduction of both excessive exchanges and routing load in IoT, specifically in RPL approach. In the proposed method of this paper, through exerting graph degree restriction on each parent node, the exchange rate was reduced as far as possible to a cogent extent. 

Furthermore, through the utilization of learning automata, packets belonging to similar directions were aggregated toward network root, and the time consumption was managed in terms of the data exchange rate. As a result, this approach yielded in the more intelligent formation of the network graph, and more efficient load balancing in the network in both simulation and practical environments. The accrued results from both simulations and practical tests results confirmed the remarkably superior performance of the proposed method in terms of energy consumption, control overhead, and root access delay as compared to the previously proposed methods.

## Figures and Tables

**Table 1:** List of variables used in the paper.

Variables	Definition	Variables	Definition
*G*	Graph	DIS	DODAG Information Solicitation
*V*	V is a set of vertices	DAO-Ack	Destination Advertisement Object Acknowledgement
*E*	E is a set of edges	{α, β, p, T}	Automata’s operations
*S*	Set of nodes	α	Action of Automata
*B*	Border routers	β	Input set of automata
*u, v*	Node in graph	*c*	Penalties of Automata
*p*	Parent node	*d*	Bonuses of Automata
*p’*	Alternative parent node	p	Probability of Automata
DODAG	Destination Oriented Directed Acyclic Graph	T	Learning Algorithm
GeoRank	A geographic routing approach for RPL	lbl−indicator	Aggregation label
Root	Root node of graph (Sink)	*F*	Aggregation Function
Rank	Number hops of Root node in a DODAG	$P_{Agg}$	Probability of Aggregation by node
OF	Objective Function	$P_{AN}$	Number of data packets aggregated by previous nodes
DPP	DODAG Preferred Parent	$P_{AM}$	Number of data packets not being aggregated by previous nodes
DRL	DODAG Root List	*RS*	Reinforcement Signal
*k*	Constant K is degree of graph	Rate j	Rate of input packets
DIO	DAG Information Object	$N_{PK_{i}}$	Number of aggregated data packets in i
DAO	Destination Advertisement Object	PDR	Packet Delivery Ratio

**Table 2:** Specifications of sensor nodes Gloriot in Cooja environment.

Part	Description
Micro	STM32f405-ARM32-bit-Cortex-M4-CPU
Flash	Up to 1 Mbyte
LP Operation	Sleep, Stop and Standby modes VBAT supply for RTC, 2032 bit backup- registers +optional 4 KB backup SRAM
Radio	TI CC2520
Routing Level	RPL based on border router
Network Layer	IPv6 with 6LoWPAN standards 802.15.4
Application Layer	GLORIOT-Interface + COAP
Battery Level	Battery holder for 2 AAA batteries
Sensors	Sensors: temperature/humidity(SHT15)
Sensor Port	Interfaced with the IRMote-CC2520
Radio Rate	30 m −1 dBm in simulation and 2 m in experimental
Propagate Model	Unit Disk Graph Model
Number of Nodes	50 randomly-deployed nodes
Node Position	Fixed without mobility
Sink/Root node Position	X/2, Y0
Warming	120 s
Data Generating	Every 20 s and 30 s UDP packet
Simulation time	2 h

**Table 3:** Results of practical tests comparing the proposed method and other approaches in indoor and outdoor environments.

Protocol	Sent	Receive	Drop	Packet Delivery Ratio(%)	Aggregated (%)
RPL(Indoor)	81,874	75,313	6561	91.9	0
RPL(Outdoor)	81,430	77,987	3443	95.7	0
BD-RPL(Indoor)	83,326	80,334	2992	96.4	0
BD-RPL(Outdoor)	81,450	79,004	2446	96.9	0
M-RPL(Indoor)	78,830	73,369	5461	93.1	6.78
M-RPL(Outdoor)	77,329	73,650	3679	95.2	9.83
A-RPL(Indoor)	80,993	78,132	2861	96.4	11.03
A-RPL(Outdoor)	82,584	79,931	2653	96.7	13.26
LA-RPL(Indoor)	83,718	82,665	1053	98.7	34.11
LA-RPL(Outdoor)	82,880	82,151	729	99.1	37.7
